# The Renin-Angiotensin System Modulates Inflammatory Processes in Atherosclerosis: Evidence from Basic Research and Clinical Studies

**DOI:** 10.1155/2009/752406

**Published:** 2009-04-14

**Authors:** Fabrizio Montecucco, Aldo Pende, François Mach

**Affiliations:** ^1^Division of Cardiology, Foundation for Medical Researches, University Hospital of Geneva, 64 Avenue Roseraie, 1211 Geneva, Switzerland; ^2^Clinic of Internal Medicine I, Department of Internal Medicine, University of Genoa 16132, Genoa, Italy

## Abstract

Recent evidence shows that the renin-angiotensin system is a crucial player in atherosclerotic processes. The regulation of arterial blood pressure was considered from its first description of the main mechanism involved. Vasoconstriction (mediated by angiotensin II) and salt and water retention (mainly due to aldosterone) were classically considered as pivotal proatherosclerotic activities. However, basic research and animal studies strongly support angiotensin II as a proinflammatory mediator, which directly induces atherosclerotic plaque development and heart remodeling. Furthermore, angiotensin II induces proatherosclerotic cytokine and chemokine secretion and increases endothelial dysfunction. Accordingly, the pharmacological inhibition of the renin-angiotensin system improves prognosis of patients with cardiovascular disease even in settings of normal baseline blood pressure. In the present review, we focused on angiotensin-convertingenzyme (ACE) inhibitors, angiotensin II receptor blockers (ARBs), and renin inhibitors to update the direct activities of the renin-angiotensin system in inflammatory processes governing atherosclerosis.

## 1. Introduction

**Figure 1:**
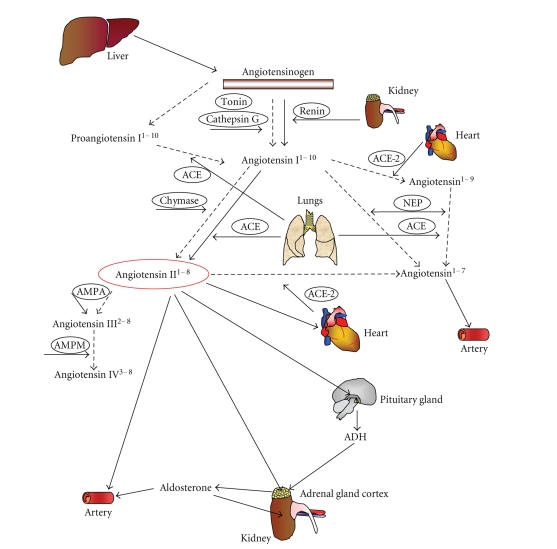
Expanded renin-angiotensin-aldosteron system. Recently, the identification of new angiotensins with different activities increased the complexity of this hormonal axis. In addition to the crucial activities of the liver, kidney, lung, adrenal gland cortex, and pituitary gland, the heart also influences this system. ACE: angiotensin converting enzyme; ACE-2: angiotensin converting enzyme 2; NEP: neutral endopaptidase; AMPA: aminopeptidase A; AMPM: aminopaptidase M.

**Figure 2:**
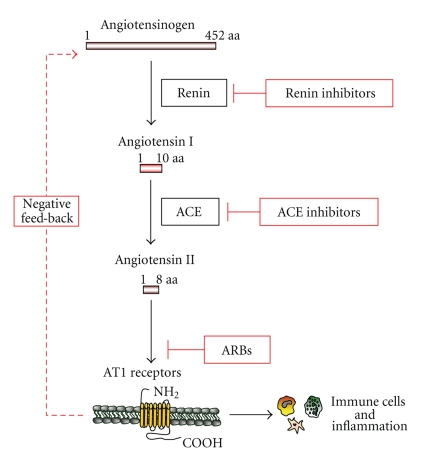
Simplified view of renin-angiotensin pathway and its pharmacological inhibition. Renin inhibitors, ACE inhibitors, and ARB modulate angiotensin activities in inflammatory processes. AT_1_ receptors, which are expressed in immune cells, have been shown to trigger inflammatory pathways.

**Figure 3:**
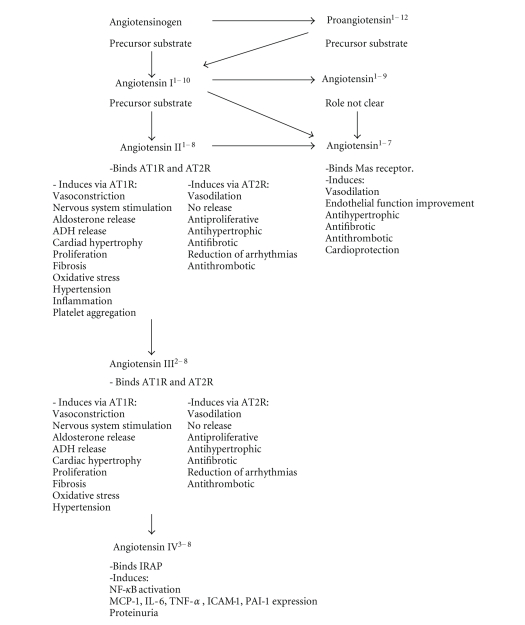
Revisited functions of the renin-angiotensin axis. Recent studies support that angiotensins influence several processes, including inflammation. AT_1_R: angiotensin type 1 receptor; AT_2_R: angiotensin type 2 receptor; IRAP: insulin-regulated aminopeptidase; Mas (mas oncogene) receptor.

Atherosclerosis is a chronic inflammatory disease, which involves vascular cells, immune system, and several organs [1]. Although leukocytes, endothelial and smooth muscle cells have been shown to play a crucial role in atherosclerotic inflammation, recent evidence also supports a direct activity for the liver, lung, heart, kidney, adipose tissue, adrenal, pancreatic, pituitary, and sex glands [2]. These organs produce several soluble inflammatory mediators, which orchestrate vascular and immune cell functions. Although cytokines, chemokines as well as growth factors have been shown to modulate inflammatory processes, recent studies suggest new inflammatory activities for endocrine hormones [3, 4]. The renin-angiotensin system could serve an important role in promoting inflammation [4, 5]. However, despite its first description by Tigerstedt and Bergman over a century ago [6], the role of these hormones in inflammatory processes is still unclear. The recent identification of new angiotensins and the different roles of angiotensin and renin/prorenin receptors increased the complexity of this system, suggesting that further investigations are needed to better understand the role of renin-angiotensin axis in inflammation (Figure 1) [7–9]. Furthermore, the description of the angiotensin-converting enzyme (ACE) 2 and its main product (angiotensin^1–7^) raised some controversies [10, 11]. ACE 2 and angiotensin^1–7^ levels are not influenced by ACE inhibitors or angiotensin II receptor blockers (ARBs). On the other hand, the “negative feed-back” regulating plasma renin activity is modulated by these drugs [12] (Figure 2). ACE 2 and angiotensin^1–7^ rather appear to be upregulated by these drugs maily in the myocardium and kidney [13, 14]. ACE 2 is also highly expressed in hypothalamus and aorta, and it is considered as a possible modulator of the renin-angiotensin system [15]. In particular, both ACE 2 and angiotensin^1–7^ may counterbalance excess of activity of the “classical” renin-angiotensin system (Figure 3). Angiontenin II has been detected also in peripheral tissues (such as aortic tissue), suggesting a possible role of the local renin-angiotensin system in atherosclerosis [16]. Both local and circulating angiotensin II exert their activities through the binding to angiotensin II type 1 (AT_1_) or type 2 (AT_2_) receptors. AT_1_ receptor is widely expressed on different cell types involved in atherosclerogenesis [17]. AT_2_ receptors are ubiquitously expressed in foetus and dramatically fall in the first few hours after birth [18]. Recently, a local renin-angiotensin system characterized by the expression of both AT_1_ and AT_2_ receptors has been also shown in adipose tissue [19]. Furthermore, the rediscovery of the “intracellular” activity of angiotensin II as a major factor involved in cardiac remodeling suggested new possible investigation fields [20–22]. The present review will be focused on evidences from basic research studies and clinical trials, investigating the role of the “revisited” renin-angiotensin system [7] and its pharmacological inhibitions in atherosclerotic inflammatory processes (Figure 2). 

## 2. ACE Inhibitors, ARBs, and Renin Inhibitors in Atherosclerotic Inflammatory Processes: Basic Research and Animal Studies

In the last decades, basic researches have strongly suggested that the renin-angiotensin system blockade exerts potent antiatherosclerotic effects, not only through the antihypertensive pathway but also through anti-inflammatory, antiproliferative, and antioxidant properties [23]. Among these hormones, angiotensin II is considered as the main proatherosclerotic mediator. Angiotensin II regulates not only adhesion molecule (VCAM-1, ICAM-1, P-selectin) expression but also cytokine, chemokine, and growth factor secretion within the arterial wall [24]. On the other hand, the renin-angiotensin system can modulate the activation of complement system in both atherosclerosis and renal injury [25–27]. This inflammatory cascade activates the vascular inflammatory response by increasing inflammatory cell recruitment to intima. Recruited cells can produce angiotensin II (intracellular angiotensin system), resulting in a positive feedback response, which can maintain this inflammatory vicious circle. In humans, an analysis of both ruptured and hypercellular plaques demonstrated high levels of ACE in macrophages. Accordingly, little or no ACE was found in areas with only fibrotic plaques [28, 29]. These data suggest that ACE may be associated to atherosclerotic plaque development and vulnerability through the direct regulation of inflammatory cells. Furthermore, angiotensin II favors the intraplaque recruitment of monocytes and lymphocytes [30] and directly enhances TNF-*α*, IL-6 and cyclooxygenase-2 expression in atherosclerotic arteries [31]. Angiotensin II-mediated effect could be potentitated by C-reactive protein (CRP) through the upregulation of AT_1_ receptor expression in vascular smooth muscle cells [32]. Angiotensin II has been also shown to increase LDL oxidation in macrophages [33, 34], oxLDL receptor (LOX-1) expression in endothelial cells [35], superoxide and metalloproteinase production, and lipid peroxidation [36]. In addition, the inactivation of nitric oxide (NO) and prostacyclin (PGI_2_) has been also observed in the presence of angiotensin II [37–40]. The binding between angiotensin II and AT_1_ receptor induced proinflammatory effect mainly through the down-stream activation of intracellular signaling cascade, which involves nuclear factor-kappaB (NF-*κ*B) activation [41–43]. The activation of NF-*κ*B pathway increases hypertension-induced renal damage [44]. However, Henke et al. clearly showed that, despite the development of high blood pressure, in vivo NF-*κ*B pathway suppression in endothelial cells reduced hypertension-induced renal damage in mice with endothelial cell-restricted NF-*κ*B superrepressor IkappaBalphaDeltaN overexpression [45]. Accordingly, the activation of NF-*κ*B pathways is also crucial in atherogenesis and macrophage activation/survival [46–48]. Therefore, angiotensin II through the activation of NF-*κ*B pathway could directly increase atherosclerotic inflammation. The majority of the direct proinflammatory effects induced by angiotensin II have been shown in studies with selective AT_1_ receptor blockers. Conversely, Kato et al. showed that renin-angiotensin system-activated transgenic mice receiving bone marrow transplantation from AT_1_a knockout (KO) mice. These transgenic animals displayed accelerated atherosclerosis and mortality [49]. The lethal effect was mainly mediated by AT_1_a KO macrophages that overexpressed a number of genes involved in atherogenesis and exhibited a greater uptake of modified lipoproteins [49]. Given the controversial role of AT_1_ receptors, further investigations are needed. Less is known about AT_2_ receptors. They are mainly localized in cardiac interstitial fibroblasts and are capable of binding not only angiotensin II but also other angiotensins, including angiotensin III. AT_2_ receptors also signal through NF-*κ*B-mediated pathways but they may counterbalance AT_1_ receptor-mediated effects through the activation of phosphatases rather than kinases [50–52]. AT_2_ receptor pathways increase bradykinin production and NO synthase activity in endothelial cells [50]. AT_2_ receptor activation also inhibits growth of cultured vascular smooth muscle cells and cardiac myocytes [51, 52]. On the other hand, the selective AT_2_ receptor blockade has been shown to inhibit in vivo medial smooth muscle hypertrophy and fibrosis in hypertensive rats [51]. These controversial results suggest that also the role of AT_2_ receptors is still not clear. The renin-angiotenin system also influences inflammatory mediators involved in the coagulation cascade. In particular, this hormonal axis inhibits fibrinolysis and enhances thrombosis by increasing plasminogen activator-1 production in endothelial and vascular smooth muscle cells [53–55] and by activating platelets [56]. The renin-angiotensin system also stimulates platelets to release thromboxane A2 and platelet derived growth factor [54] and increases tissue factor levels in atherosclerotic plaques in acute coronary syndromes [57]. These basic research studies suggested that the pharmacological inhibition of the renin-angiotensin system may be of benefit against atherosclerotic inflammatory processes. In fact, ACE inhibitors or ARBs do not modulate exclusively kidney and arterial cell functions [58]. Inflammatory cell, adipocyte, and cardiomyocyte functions are directly regulated by these drugs [59–63]. Animal models partially confirmed these encouraging results. Two decades ago, the first preclinical studies in vivo showed that ACE inhibitors had not only blood-pressure-lowering properties [64] but also direct protective effects on endothelium and atherogenesis [65]. At an early stage of atherosclerosis, the treatment with different ACE inhibitors reduced endothelial dysfunction in atherogenic diet-fed [66] or hyperlipidemic rabbits [67]. Quinapril reduced macrophage infiltration in atherosclerotic lesions in femoral arteries in rabbits through the direct inhibition of macrophage chemoattractant protein (MCP)-1 expression. Accordingly, angiotensin II itself increased MCP-1 expression in atherosclerotic lesions, thus contributing to macrophage infiltration [68]. The crucial role of the renin-angiotensin system in inflammatory processes regulating atherosclerosis was also observed in other animal models prone to develop atherosclerosis [69–74]. In these studies, various ACE inhibitors at doses comparable to those used clinically reduced atherosclerotic lesions independently of blood pressure. This was suggested by two independent findings: (1) the use of other antihypertensive drugs did not produce similar results [75]; (2) ACE inhibitors reduced atherosclerosis without altering blood pressure [69]. The beneficial effects of the renin-angiotensin pharmacological inhibition have been also observed in animal models of hypertension. The most used model was the “stroke-prone” strain (SHR-SP) rats. Treatment with ramipril in the pre-hypertensive phase in SHR-SP rats strongly reduced mortality and improved left-ventricular hypertrophy, cardiac and endothelial functions, and metabolism [76]. The administration of ACE inhibitors in the later phases of hypertension in SHR-SP rats also decreased mortality [77], suggesting that ACE inhibitors reduce cardiovascular risk and atherosclerosis in animals in different stages of cardiovascular disease. These benefits are confirmed by the majority of the published studies. However, some authors have also demonstrated that lowdose of trandolapril did not reduce both blood pressure and atherosclerosis in hyperlipidemic rabbits [78]. Similarly to ACEinhibitors, ARBs reduced blood pressure and atherosclerosis in different animal models [79–82]. However, differently from ACE inhibitors, the protective effect of ARBs was observed at both high and lowdoses. Although some authors did not confirm ARB-mediated benefits at low doses [83], a possible dose-dependent impact on atherogenesis, not only mediated by blood pressure lowering, is strongly suggested. No data are available on the use of ACE inhibitors and ARBs on atherogenesis in transgenic rats with inducible angiotensin II (Ang II)-dependent hypertension (TGR[Cyp1a1-Ren2]), two kidney-one clip (2K-1C) hypertension rats, or hypertensive double transgenic mice (R+/A+) that overexpress both human renin (R+) and human angiotensinogen (A+). Few evidences are available about the use of ACE inhibitors or ARBs on mouse models with local (intracardiac) or systemic high angiotensin II [84, 85]. However, these studies were not focused on atherosclerosis. Therefore, further studies are needed to clarify the role of ACE inhibitors and ARBs in atherosclerosis in animal models with high angiotensin II levels. In March 2007, the US Food and Drud Administration approved a new renin-angiotensin blocker (aliskiren, a direct renin inhibitor) for the treatment of hypertension in humans without renal dysfunction. Renin inhibition blocks angiotensin I generation with the consequent suppression of angiotensin II as well as angiotensin peptide formation. Preclinical studies strongly supported the antihypertensive efficacy and safety of aliskiren [86]. Recent evidence also suggested a possible direct role of renin inhibitors to reduce atherosclerotic inflammation [87–89]. In a double-trangenic rat model (dTGR), overexpressing human renin and human angiotensinogen genes, aliskiren reduced cardiac hypertrophy, fibrosis, inflammation, and inducibility of arrhythmias [90] and reversed already established cardiac and renal damage [91]. The benicial effects of renin inhibition on organ damage are partially due to the suppression of hypertension. The blockade of direct proinflammatory activities of angiotensin II and angiotensin peptides represents a crucial mechanism to reduce atherosclerosis. In fact, in the same dTGR rat model, aliskiren and ARB losartan also reduced albuminuria and expression of inflammatory mediators, such as TNF-*α*, C-reactive protein (CRP) and complement C1q, C3, C3c, and C5b-9 in comparison with untreated controls [25]. Treatment with aliskiren has been also shown to protect against endothelial dysfunction and atherosclerosis in Watanabe heritable hyperlipidemic rabbits [89] as well as ApoE deficient [87] or LDL receptor deficient [92] mice.

## 3. ACE Inhibitors, ARBs, and Renin Inhibitors in Atherosclerotic Inflammatory Processes: Clinical Trials

At the beginning of the nineties, Dzau and Braunwald proposed the concept of the cardiovascular continuum in humans [103]: cardiovascular disease can be seen as a pathophysiologic cascade induced by the presence of risk factors, such as hypertension, hypercholesterolemia, diabetes mellitus, and smoking. These conditions can produce well defined stages, such as endothelial dysfunction, atherosclerosis, and target organ damage, followed ultimately by the clinical syndromes (heart failure, stroke, and end-stage renal disease) and eventually death. Experimental evidence clearly suggests a key role of the renin-angiotensin system and the induced inflammatory processes at all stages of this continuum and consequently a strong rationale for its blockade in order to prevent cardiovascular events [23]. The possibility of a positive effect of the renin-angiotensin blockade at the early stages of the cardiovascular continuum, that is, the endothelial dysfunction, was specifically addressed by some clinical studies. Although the complexity of the methodology applied to these investigations did not allow the recruitment of a very large number of patients, the results were clearly supportive about the role of the renin-angiotensin inhibition in the reversal of the endothelial dysfunction. Early evidence came from the TREND study [104], which showed that angiotensin-converting enzyme (ACE) inhibition with quinapril improves endothelial function of the coronary arteries. Similar results were obtained in the coronary circulation with the ARBs: valsartan improved basal nitric oxide production and release in hypertensive patients as compared to diuretic-treated subjects, despite similar blood pressure decrease [105]. The endothelial function was evaluated also in both the peripheral [106] and the renal circulation [107], always showing a consistent improvement exerted by the renin-angiotensin blockade. In addition, in a small group of hypertensive patients, resistance arteries obtained from subcutaneous biopsies were studied before and after 1 year of treatment with either an ARB (losartan) or a *β*-blocker (atenolol); basal measurements were compared to those of normotensive controls [108]. Despite similar reductions in blood pressure, losartan normalized acetylcholine-dependent vasodilation and reduced media/lumen ratio. Whereas different ARBs exert their effects on endothelial function in a similar way (through AT_1_ receptor antagonism) for ACE inhibitors we have to consider the presence of both plasma ACE, which regulates blood pressure, and tissue ACE, which is involved in the regulation of tissue inflammation, fibrosis, and hypertrophy [109]. In BANFF study, for example, an ACE inhibitor with low activity at the tissue level, enalapril, was not able to affect endothelial function [110]. More recently the TRENDY study tried to compare an ARB, telmisartan, and an ACE inhibitor, ramipril, in terms of improvement of the renal endothelial function [107]: no significant differences between the two drugs were observed although the ARB seemed to be a little more efficient. The demonstration of the prognostic significance of endothelial dysfunction was obtained from studies where it was possible to find an inverse association between the acetylcholine-stimulated forearm blood flow increase and the cumulative incidence of cardiovascular events [111]. Two other surrogate parameters, which have been evaluated in clinical studies extensively, are the circulating inflammatory markers and the extension of the vascular damage (carotid intima-media thickness [IMT], coronary circulation, and volume of the atherosclerotic plaques). Although ACE inhibitors reduce blood levels of inflammatory cytokines in vivo [102], this issue has been addressed more in depth for ARBs [112]. Table 1 lists a series of clinical studies, in which ACE inhibitors and ARBs reduce serum levels of inflammatory markers in different diseases [93–102]. As for vascular structure, the less invasive way to evaluate the possible atherosclerotic changes is the ultrasound determination of the carotid IMT. In the SECURE trial, a significant decrease in the progression slope of mean maximal IMT by 0.04 mm was observed in the active arm as compared to placebo [113]. These results were not confirmed by another study, the PART-2 trial, with the same active drug and the same parameter [114], and also by two studies (QUIET and SCAT) with a coronary angiographic evaluation [115, 116]. More recently intravascular ultrasound (IVUS) was used in a substudy of the CAMELOT trial, which compared the effects of 3 different treatments on atherosclerosis progression: amlodipine, a calcium-antagonist, showed no progression; enalapril, an ACE inhibitor, a trend toward progression, which was more evident in the placebo group [117]. Also the effects of ARBs were evaluated at the vascular levels: in a substudy of the LIFE trial, losartan, an ARB, but not atenolol, a *β*-blocker, induced a regression of the carotid artery hypertrophy in hypertensive subjects [118]. The first successful clinical application of the experimental observations about the role of the renin-angiotensin in the cardiovascular pathophysiology was the demonstration of the ACE inhibitors as an undisputed treatment in patients with congestive heart failure or coronary artery disease (CAD) and concomitant left ventricular dysfunction, all clinical syndromes characterized by a strong activation of the renin-angiotensin system [119]. These results were subsequently confirmed by trials with ARBs [120]. The first studies (SAVE and SOLVD) demonstrated that these drugs reduced both mortality rate and risk of ischemic events [121]; moreover in SAVE the effect of the ACEinhibition by captopril was found to be independent of the degree of left ventricular dysfunction. These data suggested a primary anti-ischemic effect of the ACEinhibitors. Therefore a subsequent step was proposed to demonstrate a significant positive effect of the renin-angiotensin blockade in subjects at high risk for cardiovascular events but without left ventricular dysfunction. The HOPE study was the first randomized controlled trial that reached this goal: in high-risk patients ramipril was able to induce an important 22% risk reduction of composite cardiovascular death, MI, or stroke compared to placebo [122]. High-risk patients were defined as those with evidence of vascular disease (CAD, stroke, peripheral vascular disease) or diabetes plus one other cardiovascular risk factor (hypertension, low high-density lipoprotein levels, elevated total cholesterol levels, smoking, microalbuminuria). The analysis of the results induced intense debate about the role of blood pressure decrease per se in the observed benefits [123]. The results in the HOPE study and in the subsequent similar trials with other ACEinhibitors occurred in a population of patients already receiving standard medical therapy, including platelet inhibitors, lipid-lowering therapy, and *β*-blockers. Two large trials (EUROPA and PEACE) were performed to confirm HOPE study results with the same class of renin-angiotensin antagonists [124, 125]. The former was successful in demonstrating a similar (20%) relative reduction of cardiovascular risk (primary end point of cardiovascular death, MI, or cardiac arrest); instead the latter was not able to show significant differences between treatment groups (ACEinhibitor versus placebo) in the primary end point, a composite of death resulting from cardiovascular causes, nonfatal MI, or revascularization. Several hypotheses have been put forward to explain these discrepancies [126]: the main reason could be the healthier conditions of the PEACE patients with respect to the patients enrolled in the other trials and therefore the difficulty for the active treatment to demonstrate clear positive effects on the outcomes. However a meta-analysis of the three placebo-controlled trials demonstrated a significant effect of ACE inhibition on the occurrence of all-cause mortality, cardiovascular mortality, nonfatal MI, stroke, heart failure, and coronary bypass surgery [127]. The same approach has been performed with an ARB in the TRANSCEND study [128]. The results have shown that the active treatment (telmisartan) was not superior to placebo in the prevention of cardiovascular events, primary composite end point represented by cardiovascular death, MI, stroke, or admission to the hospital for heart failure events. Ripley and Harrison suggest that these partially unexpected data could be explained by the differences in patient number, event rates, and the use of other life-saving drugs between TRANSCEND and HOPE studies [129]. However these results confirm the difficulty to demonstrate a significant effect of the renin-angiotensin blockade in the cardiovascular prevention beyond the blood pressure control. At present, the only suggestion of a therapeutical action which could be independent from the changes in blood pressure levels derives from the LIFE study [130]. In this large multicenter trial patients with left ventricular hypertrophy were randomized to receive treatment based on an ARB (losartan) or a *β*-blocker (atenolol): the composite primary end point of death, MI, and stroke was reduced by 13% with the ARB-based treatment compared with the *β*-blocker-based treatment in presence of a similar amount of blood pressure decrease. Another important issue is the mechanism of action of the different classes of renin-angiotensin blockers. If angiotensin II is the key player in the inflammatory processes in cardiovascular disease, we have many pharmacological ways to inhibit its synthesis; in addition the different classes of drugs demonstrate other effects, possibly related to a therapeutic gain (so called pleiotropic actions). In fact it is well known that ACE inhibitors are able to reduce the breakdown of bradykinin, and this molecule can cause the most frequent untoward effects of these drugs (cough, angioedema) but it is believed also as an important contributor to the protective cardiovascular effects exerted by them [131]. On the other hand, ARBs significantly increase angiotensin II levels, as a consequence of the antagonism at the AT_1_ receptor site. The possible role of the AT_2_ receptor stimulation in the beneficial therapeutic effects of ARB remains a fascinating hypothesis [132, 133]. These pharmacological differences could explain the possible better results obtained with ACE inhibitors in terms of prevention of coronary events and with ARBs in terms of prevention of ischemic strokes [134] in comparison with the direct competitors for renin-angiotensin blockade. This therapeutic hypothesis has been verified by a systematic review of the available clinical data about the two classes of drugs [135] and by the recently published ONTARGET trial, a very large multicenter randomized trial in which the patients were treated with an ACE inhibitor (ramipril), an ARB (telmisartan), or the combination of the two drugs [136]. After a median follow-up of 56 months, the occurrence of the primary outcomes, consisting of death from cardiovascular causes, MI, stroke, or hospitalization for heart failure, was not significantly different in the ramipril and telmisartan groups, although the ARB was better tolerated. There were trends slightly favoring the ACE inhibitor for MI prevention and the ARB for stroke prevention but these differences did not reach statistical significance. The other issue addressed by the trial, the clinical role of the combined renin-angiotensin blockade, brought a word of caution about this strategy since more adverse events were observed [137, 138]. Although in conditions of renin-angiotensin hyperactivation, such as advanced heart failure, and of marked proteinuria the double blockade can still exert beneficial effects, other recent studies confirmed the possible risk of the combination in both a cardiological and a nephrological setting [139–141]. In 1957, Skeggs et al. suggested another possible approach to pharmacologically inhibit the renin-angiotensin system [142]. Renin inhibition was indicated as the preferred step to reduce angiotensin II effects. The discovery of prorenin receptor constitutes an additional reason to develop a new class of renin inhibitors [9]. An ambitious plan of primary and secondary prevention trials has begun in order to demonstrate possible advantages of the treatment with aliskiren alone or in combination with other renin-angiotensin blockers in patients with hypertension. At present, the effects independent of antihapertensive activity of aliskiren have been shown by one clinical trials focused on end-organ damage. In aliskiren in the evaluation of proteinuria in diabetes (AVOID) trial, the treatment with aliskiren reduced proteinuria independently of blood pressure [143]. Other clinical trials have been started to investigate the possible benefits of aliskiren in cardiac remodeling after myocardial infarction (AVANT GARDE, ASPIRE) and diabetic nephropathy (ALTITUTE) [144]. Therefore, in the next future, further clinical evidence will be available to confirm these preliminary anti-inflammatory and antiatherosclerotic effects of aliskiren in humans.

## 4. Conclusions

The inhibition of the renin-angiotensin system represents a pivotal approach for reducing atherosclerosis and its dramatic complications, such as stroke and myocardial infarction (MI). ACE inhibitors and ARBs are well-established pharmacological tools in both primary and secondary prevention of atherosclerotic cardiovascular disease. Emerging evidence shows that their beneficial effects are not only due to blood pressure lowering but also due to a direct anti-inflammatory activity. Further studies are needed to better understand this promising investigation field, with particular interest for the promising results with the new renin inhibitor treatment.

## Figures and Tables

**Table 1:** Clinical studies evaluating effects of RAS blockade on circulating inflammatory markers. When two active drugs are administered, the effects demonstrated are with respect to basal values; when an active deug and placebo are used, the comparisons are between the two arms. CABG: coronary artery bypass grafting; ACEI: ACE inhibitors.

Studies	Patients	Clinical conditions	Drugs	Main effects	
Sheth et al. [93]	107	Chronic heart failure	Lisinopril versus Omapatrilat	↑ IL-10 (Omapatrilat)	
				= IL-6	

Jilma et al. [94]	32	Essential hypertension	Enalapril versus Losartan	↓ E-selectin	Enalapril
				↓ ICAM-1	
				↓ VCAM-1	
				↓ MCP-1	

Koh et al. [95]	45	Essential hypertension	Candesartan versus Placebo	↓ MCP-1	
				↓ TNF-*α*
				= CRP

Di Napoli and Papa [96]	507	Ischemic stroke	ACEI versus Other hypotensive drugs	↓ CRP (ACEI)	

Tsikouris et al. [97]	30	Acute myocardial infarction	Quinapril versus Enalapril	↓ CRP (Quinapril)	

Schieffer et al. [98]	48	Coronary artery disease Essential hypertension	Enalapril versus Irbesartan	↑ IL-10	Both
				↓ MMP-9	
				↓ IL-6	Irbesartan
				↓ CRP	

Fliser et al. [99]	199	Essential hypertension and/or Vascular disease Diabetes mellitus LDL-C >150 mg/dL	Olmesartan versus Placebo	↓ CRP	
				↓ TNF-*α*	
				↓ IL-6	
				↓ MCP-1	

Trevelyan et al. [100]	45	Angina pectoris awaiting CABG	Enalapril or Losartan versus Control	↓ IL-1ra	
				↓ IL-6	
				= IL-10	
				= IL-8 n.d.	

Tikiz et al. [101]	45	Rheumatoid arthritis	Quinapril versus Placebo	= CRP	
				= TNF-*α*	
				= IL-1*β*	
				= IL-6	

Krysiak and Okopień [102]	90	Coronary artery disease	Perindopril or Enalapril versus Placebo	↓ MCP-1	Both
				↑ IL-10	
				↓ CRP	(Perindopril)
